# Pantoprazole-Associated Thrombocytopenia: A Literature Review and Case Report

**DOI:** 10.7759/cureus.22326

**Published:** 2022-02-17

**Authors:** Alexander T Phan, Alan W Tseng, Mohammad W Choudhery, Jelena B Makar, Cyrus Nguyen, Farbod Farmand

**Affiliations:** 1 Internal Medicine, Arrowhead Regional Medical Center, Colton, USA; 2 Emergency Medicine, Arrowhead Regional Medical Center, Colton, USA

**Keywords:** platelet count (plt), proton-pump inhibitor, clinical hematology, pantoprazole, drug-induced thrombocytopenia

## Abstract

Proton-pump inhibitors (PPIs) are commonly utilized in the treatment of upper gastrointestinal bleeds (UGIBs) due to their ability to stabilize blood clot formation. PPIs have been shown to reduce rebleeding after endoscopic hemostasis and reduce signs of bleeding at index endoscopy. While PPIs are well-tolerated and commonly administered to patients suffering from acute UGIBs, significant adverse effects may occur. Patients have reported various mild systemic symptoms during short-term PPI use, including headache, rash, dizziness, nausea, abdominal pain, flatulence, constipation, and diarrhea. In general, serious side effects of PPIs tend to be mild during treatment periods under two weeks; however, as the treatment duration increases, side effects have been observed to increase in frequency and severity. PPI-induced thrombocytopenia is an exceedingly rarely reported adverse reaction that remains largely unstudied due to the dearth of patient cases. This adverse effect continues to be a diagnosis of exclusion, and there are no current evidence-based recommendations to approach this complication. Thrombocytopenia increases the risk of rebleeding and hemodynamic instability, which may be devastating to patients suffering from UGIBs. Here, we present a case of thrombocytopenia that began after the introduction of pantoprazole in the setting of a UGIB. The thrombocytopenia resolved promptly after cessation of the medication. We highlight this case to increase awareness of this rare finding given the lack of recommendations for short-term PPI-induced thrombocytopenia.

## Introduction

Proton-pump inhibitors (PPIs) are a class of drugs that are widely used for the treatment of a variety of gastric acid-related disorders, including peptic ulcer disease (PUD) and gastroesophageal reflux disease (GERD) [1]. They are also routinely used in the treatment of upper gastrointestinal bleeds (UGIBs) [2]. Inhibition of gastric H+/K+ ATPase proton pumps helps to not only reduce pain and progression of conditions like GERD and PUD but also by increasing serum pH, these drugs increase platelet aggregation and plasma coagulation, thereby stabilizing blood clot formation [3]. PPIs have been shown to reduce rebleeding after endoscopic hemostasis and, when started before endoscopy, reduce signs of bleeding at index endoscopy [4-5].

PPIs are commonly prescribed medications that are considered to be generally safe and well-tolerated. In fact, these drugs account for approximately 95% of all acid-suppressing medication prescriptions due to their effectiveness and side-effect profile [6]. While these drugs have significant benefits to patients suffering from an acute UGIB, significant adverse effects can occur during both short and long-term therapy [6]. The adverse effects of long-term use that have been reported include increased risk of enteric infection, chronic atrophic gastritis, drug-induced lupus, acute interstitial nephritis, thrombocytopenia, and malabsorption of various nutrients like iron, vitamin B12, and magnesium [6-12].

With the short-term initiation of PPIs in the acute setting, most adverse effects have been relatively well studied and reported [13]. Patients may report various mild systemic symptoms during short-term PPI use, including headache, rash, dizziness, nausea, abdominal pain, flatulence, constipation, and diarrhea [6]. In general, serious side effects of PPIs tend to be mild during treatment periods of two weeks or less, but as the treatment duration increases, side effects have been observed to increase in frequency and severity with long‐term use [14]. 

Despite the large body of literature documenting the short-term effects of PPIs, the effect of PPI-induced thrombocytopenia is a rarely reported adverse reaction that remains largely unstudied. To date, there are approximately 9 case reports of PPI-related thrombocytopenic events in the current literature [15-23]. As this adverse event is poorly studied and relatively rare, PPI-induced thrombocytopenia is often a diagnosis of exclusion, leading to delays in diagnosis. Even after diagnosis, clinicians may find that there are currently no evidence-based recommendations to manage this complication. Thrombocytopenia, if left untreated, increases the risk of rebleeding and hemodynamic instability, which may be devastating to patients suffering from UGIBs. Due to the potentially significant consequences of PPI-induced thrombocytopenia, the study of this rare adverse effect is important for improving the detection and prevention of serious adverse outcomes.

## Case presentation

A 66-year-old African-American female with a past medical history of end-stage renal disease (ESRD), hypertension, a history of venous thromboembolism, and chronic lower back pain presented with intractable hematemesis and nausea. She reported six episodes of bright-red, bloody emesis with blood clots and rectal pain associated with bloody stools. The patient denied abdominal pain, history of anemia, chest pain, shortness of breath, history of liver disease, history of malignancy, and constipation. On presentation, vital signs included a temperature of 97.8 F, pulse rate of 89, respiratory rate of 15, oxygen saturation of 97% on room air, and blood pressure 86/62 mmHg. The physical exam was notable for dried blood on the lips, 2+ lower extremity pitting edema bilaterally, and dry oral mucosa. The patient’s initial laboratory results are shown in Table 1, demonstrating severe anemia, decreased hematocrit, elevated red cell distribution width, elevated blood urea nitrogen, and elevated creatinine. Serum electrolytes, liver function tests, and platelet count were within normal limits (Table 1).

**Table 1:** Initial laboratory results on admission to the hospital demonstrating severe anemia, decreased hematocrit, elevated RDW, elevated BUN, elevated creatinine, moderate hypochromasia, few macrocytic cells, and adequate platelet estimate g = gram, dL = deciliter, μL = microliter, U = unit, L = liter, mEq = milliequivalent, mmol = millimole, mg = milligram, RDW = red cell distribution width, AST = aspartate transaminase, ALT = alanine transaminase, BUN = blood urea nitrogen

	Hemoglobin (g/dL)	Hematocrit (%)	Platelet (cells/μL)	RDW (%)	AST (U/L)	ALT (U/L)	Total Bilirubin (mg/dL)	Peripheral Smear Findings
Reference Values	11.5-15.5	36-46	120-360	11.0-15.0	5-40	5-40	0-1.2	n/a
Measured Values	7	23.4	193	19	14	5	0.3	Moderate Hypochromasia
								Few Macrocytic Cells
								Adequate Platelet Estimate
	Sodium (mEq/dL)	Potassium (mEq/dL)	Chloride (mEq/dL)	Carbon Dioxide (mmol/L)	BUN (mg/dL)	Creatinine (mg/dL)	Calcium (mg/dL)	
Reference Values	135-148	3.5-5.5	98-110	24-34	8-20	0.5-1.5	8.4-10.2	
Measured Values	137	4.3	95	21	49	7.7	8.4	

She was given 1L of normal saline for hypotension and transfused 1 unit of packed red blood cells for her severe anemia. A nasogastric tube was placed to suction, which produced coffee ground-like output. Her home dose of anticoagulation was stopped, and she was started on intravenous (IV) pantoprazole 40 mg twice daily for the treatment of a presumed upper gastrointestinal bleed. Gastroenterology specialists were consulted and performed an esophagogastroduodenoscopy (EGD), which revealed severe esophagitis with oozing and evidence of a Roux-en-Y gastric bypass; however, there was no evidence of acute bleeding. After the EGD, the patient was continued on pantoprazole 40 mg IV twice daily for severe esophagitis.

**Figure 1:**
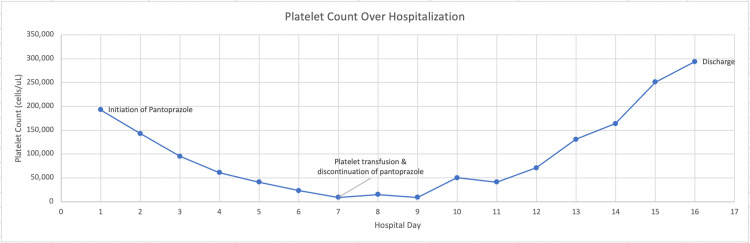
Platelet count trend of the patient over the hospital course A negative trend in platelet count was noted after initiating pantoprazole therapy, and platelet counts returned to normal following platelet transfusion and discontinuation of pantoprazole. X-axis = chronological numerical date of hospitalization, Y-axis = platelet count notated in cells/μL, μL = microliter

Over the subsequent days, the patient’s hemoglobin level stabilized; however, the patient’s platelet count progressively declined and she became severely thrombocytopenic on Day 7 of hospitalization, which can be seen in Figure 1. The hematology specialists were consulted and recommended evaluation for other causes of thrombocytopenia, including immune thrombocytopenic purpura (ITP), thrombotic thrombocytopenic purpura (TTP), hemolytic uremic syndrome (HUS), and disseminated intravascular coagulopathy (DIC). They also recommended transfusing platelets to maintain a goal platelet count of >20,000 cells/uL and to switch pantoprazole to famotidine due to possible PPI-induced thrombocytopenia. Consequently, pantoprazole therapy was discontinued, oral famotidine 10 mg once daily was initiated, and 2 units of platelets were transfused. Additional laboratory tests were ordered and the results did not suggest TTP, HUS, or DIC (Table 2). In the subsequent weeks after discontinuing pantoprazole, the patient’s platelet count returned to her baseline levels (Figure 1). She was ultimately discharged on oral famotidine 10 mg daily and experienced no further complications since her discharge.

**Table 2:** Laboratory results on hospital Day 7 demonstrating prolonged APTT, mildly elevated fibrinogen, elevated D-dimer, and polychromasia PT = prothrombin time, INR = international normalized ratio, APTT = activated partial thromboplastin time, mg = milligram, ng = nanogram, mL = milliliter

	PT (seconds)	INR (n/a)	APTT (seconds)	Fibrinogen (mg%)	D-Dimer (ng/mL)	Reticulocyte Count (%)	Immature Platelet Fraction (%)	Peripheral Smear Findings
Reference Values	11-15	n/a	25-36	174-482	<250	0.3-2	0.9-11.2	n/a
Measured Values	14.4	1.3	43.8	495	1445	1	7.80%	Polychromasia

## Discussion

Peptic ulcers are the most common cause of UGIBs, and PPIs are widely used for ulcer bleeding [24]. Commonly used PPIs include pantoprazole, esomeprazole, lansoprazole, and dexlansoprazole. Gastric acid suppression through the use of PPIs is strongly recommended by the American College of Gastroenterology (ACG) after endoscopic hemostatic therapy for bleeding ulcers; however, there is no clear recommendation for its use in the pre-endoscopic setting [25]. Despite a lack of guidance for PPI utilization before endoscopy, several studies have demonstrated that PPI utilization in this setting decreases the rate of further bleeding and may reduce the need for endoscopic therapy [26-29]. Although rare, PPI therapy does have an association with an increased risk of thrombocytopenia, which may increase the risk of new bleeding and exacerbate known sources of bleeding. There are only a few case reports that elucidate the relationship between PPI use and thrombocytopenia, and even fewer listing pantoprazole as the causal agent [15-23]. At this time, PPI-induced thrombocytopenia remains a diagnosis of exclusion and is exceedingly rare in incidence, as evidenced by the dearth of patient cases reported in the literature.

Thrombocytopenia is defined as a platelet count measured below the 2.5th percentile of the normal platelet distribution, which is defined as 150,000 cells/μL [30-31]. The mechanisms of thrombocytopenia include decreased platelet production, increased platelet destruction or use, platelet sequestration, and platelet dilution [30]. Some causes of thrombocytopenia include, but are not limited to, malignancy, infection, marrow suppression, congenital platelet deficiencies, ITP, TTP, DIC, mechanical destruction, hemorrhage, and medication-induced platelet reduction [30]. Thus, when a patient presents with thrombocytopenia, it is imperative that the clinician considers a broad differential diagnosis for the cause of thrombocytopenia, as this is not a specific manifestation of any single disease process.

The peripheral blood smear is a useful diagnostic tool to help clinicians in determining the cause of isolated thrombocytopenia in a patient, which may reveal pseudothrombocytopenia due to platelet clumping, abnormalities in platelet morphology that may reveal existing or newly acquired platelet disorders, or clues to indicate increased platelet consumption. In the case of our patient, a review of the peripheral blood smear did not reveal any significant abnormalities that would point to these etiologies. A bone marrow biopsy can also be helpful in the evaluation of thrombocytopenia, especially in patients where a primary hematologic disorder is suspected or if the cause of the disorder is unclear. We discussed the need for a bone marrow biopsy for our patient with our hematology and oncology specialists and weighed the risks and benefits of such a procedure. With the help of their input, it was decided to first discontinue potentially offending drugs and look for other etiologies before pursuing the biopsy. Once PPI therapy was discontinued and the patient's platelet levels normalized, the biopsy was no longer deemed necessary.

Our patient initially presented with intractable hematemesis and clinical findings consistent with a UGIB. The decision was made to initiate intravenous pantoprazole therapy for UGIB based on evidence demonstrated in clinical trials and recommendations by the gastroenterology specialist [26-29]. Initially, the patient’s platelet count was 193,000 cells/μL, but following the initiation of pantoprazole therapy, her platelet count precipitously dropped, as demonstrated in Figure 1. By Day 7 of hospitalization, the patient was noted to have severe thrombocytopenia, which prompted an urgent re-evaluation of her current medications and clinical diagnoses. We ruled out potential causes of thrombocytopenia such as infection, DIC, hemorrhage, HUS, TTP, pseudothrombocytopenia, and marrow suppression. The patient’s reticulocyte count was within normal limits, ruling out hemolytic anemias, and her immature platelet fraction was elevated in conjunction with a peripheral blood smear showing polychromasia, ruling out marrow suppression (Table 2). The remainder of the peripheral smear was unremarkable, liver function tests were within normal limits, and coagulation studies did not demonstrate evidence of DIC (Tables 1-2). Additionally, because the patient improved after platelet transfusion and discontinuation of pantoprazole, it is unlikely that ITP was the etiology of her thrombocytopenia. Physical examinations failed to reveal purpura or new signs of bleeding, and the patient was hemodynamically stable. Another differential diagnosis to consider is shock marrow, as our patient presented with hypotension on arrival to the emergency department. However, since the temporal relationship between the development and recovery of thrombocytopenia in our patient correlated with the times at which pantoprazole was administered and discontinued, we did not continue to consider shock marrow as a differential diagnosis. Consequently, the most likely diagnosis was drug-induced thrombocytopenia, secondary to pantoprazole therapy.

As Korkmaz et al. discuss, the evaluation of drug-induced thrombocytopenia (DIT) comprises four criteria: i) treatment with the accused drug caused thrombocytopenia and full and continuous recovery from thrombocytopenia occurred after discontinuation of the accused drug; ii) the accused drug was the only drug used before the onset of thrombocytopenia, or other drugs were continued or reintroduced after discontinuation of the accused drug with a sustained normal platelet count; iii) other causes for thrombocytopenia have been ruled out; iv) recurrent thrombocytopenia occurred upon reuse of the accused drug. Korkmaz et al. state that if all four criteria are met, there is a definitive diagnosis of DIT; however, if only criteria 1, 2, and 3 are met, the diagnosis of DIT is probable. In our case, pantoprazole administration was associated with the development of thrombocytopenia, and withdrawal of this agent demonstrated complete and continuous recovery of the patient’s platelet count. Although pantoprazole was not the only other medication administered prior to the development of thrombocytopenia, all the other medications the patient received were continued and did not have a known risk of thrombocytopenia. Additionally, other causes of thrombocytopenia were effectively ruled out, and re-administration of pantoprazole to satisfy criterion 4 would have been unethical. Thus, according to the aforementioned criteria, the most probable cause of the patient’s thrombocytopenia was pantoprazole therapy.

Through this process, we reached a final diagnosis of pantoprazole-induced thrombocytopenia. We immediately discontinued pantoprazole therapy and switched to renally dosed famotidine for gastric acid suppression. Over the course of the next several days, the patient’s platelet count progressively recovered (Figure 1). Findings in the current literature and our observations suggest that PPIs may cause thrombocytopenia. Clinicians should be aware of this potential adverse effect, as PPIs are very commonly prescribed to the general population. Thus, when a patient presents with thrombocytopenia in the setting of PPI therapy, clinicians should empirically halt PPI therapy if all other causes of thrombocytopenia are ruled out. As PPI-induced thrombocytopenia is solely a diagnosis of exclusion and the mechanism of this disorder is largely unknown, future studies should aim to assess the incidences of thrombocytopenia in PPI therapy and determine the mechanisms of PPI-induced thrombocytopenia.

## Conclusions

This case helps elucidate the rare situation in which PPI therapy can induce severe thrombocytopenia. The current literature only has several case studies specifically listing pantoprazole as a cause of drug-induced thrombocytopenia, which can be a cause of severe morbidity and mortality if left unaddressed. Clinicians should maintain a high degree of suspicion for PPI-induced thrombocytopenia when evaluating a patient with thrombocytopenia in the setting of PPI therapy and the absence of any other causes for thrombocytopenia. Clinicians should also re-assess medications on a daily basis to limit the duration of PPI usage according to current guidelines and determine the appropriate dosing regimen for the patient. Further research may determine the underlying mechanisms and an increased propensity for thrombocytopenia in the setting of PPI therapy.

## References

[1] Wolfe MM, Sachs G (2000). Acid suppression: optimizing therapy for gastroduodenal ulcer healing, gastroesophageal reflux disease, and stress-related erosive syndrome. Gastroenterology.

[2] Khan MA, Howden CW (2018). The role of proton pump inhibitors in the management of upper gastrointestinal disorders. Gastroenterol Hepatol (N Y).

[3] Green FW, Kaplan MM, Curtis LE, and Levine PH (1978). Effect of acid and pepsin on blood coagulation and platelet aggregation. A possible contributor to prolonged gastroduodenal mucosal hemorrhage. Gastroenterology.

[4] Chan WH, Khin LW, Chung YF, Goh YC, Ong HS, Wong WK (2011). Randomized controlled trial of standard versus high-dose intravenous omeprazole after endoscopic therapy in high-risk patients with acute peptic ulcer bleeding. Br J Surg.

[5] Dorward S, Sreedharan A, Leontiadis GI, Howden CW, Moayyedi P, Forman D (2006). Proton pump inhibitor treatment initiated prior to endoscopic diagnosis in upper gastrointestinal bleeding. Cochrane Database Syst Rev.

[6] Yibirin M, De Oliveira D, Valera R, Plitt AE, Lutgen S (2021). Adverse effects associated with proton pump inhibitor use. Cureus.

[7] Schiller D, Maieron A, Schöfl R, Donnerer J (2014). Drug fever due to a single dose of pantoprazole. Pharmacology.

[8] Das S, Ganguly A, Ghosh A, Mondal S, Dey JK, Saha I (2012). Oral pantoprazole-induced acute pancreatitis in an 11-year-old child. Ther Drug Monit.

[9] Lam JR, Schneider JL, Zhao W, Corley DA (2013). Proton pump inhibitor and histamine 2 receptor antagonist use and vitamin B12 deficiency. JAMA.

[10] McColl KE (2009). Effect of proton pump inhibitors on vitamins and iron. Am J Gastroenterol.

[11] Sampathkumar K, Ramalingam R, Prabakar A, Abraham A (2013). Acute interstitial nephritis due to proton pump inhibitors. Indian J Nephrol.

[12] Taş A (2013). Thrombocytopenia as a side effect of pantoprazole. Turk J Gastroenterol.

[13] Schenk BE, Festen HP, Kuipers EJ, Klinkenberg-Knol EC, Meuwissen SG (1996). Effect of short- and long-term treatment with omeprazole on the absorption and serum levels of cobalamin. Aliment Pharmacol Ther.

[14] Haastrup PF, Thompson W, Søndergaard J, Jarbøl DE (2018). Side effects of long-term proton pump inhibitor use: a review. Basic Clin Pharmacol Toxicol.

[15] Watson TD, Stark JE, Vesta KS (2006). Pantoprazole-induced thrombocytopenia. Ann Pharmacother.

[16] Korkmaz U, Alcelik A, Eroglu M, Korkmaz AN, Aktas G (2013). Pantoprazole-induced thrombocytopenia in a patient with upper gastrointestinal bleeding. Blood Coagul Fibrinolysis.

[17] Miller JL, Gormley AK, Johnson PN (2009). Pantoprazole-induced thrombocytopenia. Indian J Pediatr.

[18] Kallam A, Singla A, Silberstein P (2015). Proton pump induced thrombocytopenia: a case report and review of literature. Platelets.

[19] Dotan E, Katz R, Bratcher J, Wasserman C, Liebman M, Panagopoulos G, Spaccavento C (2007). The prevalence of pantoprazole associated thrombocytopenia in a community hospital. Expert Opin Pharmacother.

[20] Binnetoğlu E, Akbal E, Şen H (2015). Pantoprazole-induced thrombocytopenia in patients with upper gastrointestinal bleeding. Platelets.

[21] Zlabek JA, Anderson CG (2002). Lansoprazole-induced thrombocytopenia. Ann Pharmacother.

[22] Rudelli A, Leduc I, Traulle C, Smail A, Ducroix JP, Andréjak M, Baillet J (1993). Thrombopenia following treatment with omeprazole [Article in French]. Presse Med.

[23] Hayashibara T (1998). Hemolytic anemia and thrombocytopenia associated with anti-omeprazole antibody [Article in Japanese]. Rinsho Ketsueki.

[24] Leontiadis GI, Sharma VK, Howden CW (2005). Systematic review and meta-analysis of proton pump inhibitor therapy in peptic ulcer bleeding. BMJ.

[25] Laine L, Barkun AN, Saltzman JR, Martel M, Leontiadis GI (2021). ACG clinical guideline: upper gastrointestinal and ulcer bleeding. Am J Gastroenterol.

[26] Khuroo MS, Yattoo GN, Javid G, Khan BA, Shah AA, Gulzar GM, Sodi JS (1997). A comparison of omeprazole and placebo for bleeding peptic ulcer. N Engl J Med.

[27] Lau JY, Leung WK, Wu JC (2007). Omeprazole before endoscopy in patients with gastrointestinal bleeding. N Engl J Med.

[28] Schaffalitzky de Muckadell OB, Havelund T, Harling H (1997). Effect of omeprazole on the outcome of endoscopically treated bleeding peptic ulcers. Randomized double-blind placebo-controlled multicentre study. Scand J Gastroenterol.

[29] Hasselgren G, Lind T, Lundell L (1997). Continuous intravenous infusion of omeprazole in elderly patients with peptic ulcer bleeding. Results of a placebo-controlled multicenter study. Scand J Gastroenterol.

[30] Stasi R (2012). How to approach thrombocytopenia. Hematology.

[31] Cheng CK, Chan J, Cembrowski GS, van Assendelft OW (2004). Complete blood count reference interval diagrams derived from NHANES III: stratification by age, sex, and race. Lab Hematol.

